# Influenza A H3N2 subtype virus NS1 protein targets into the nucleus and binds primarily via its C-terminal NLS2/NoLS to nucleolin and fibrillarin

**DOI:** 10.1186/1743-422X-9-167

**Published:** 2012-08-21

**Authors:** Krister Melén, Janne Tynell, Riku Fagerlund, Pascal Roussel, Danièle Hernandez-Verdun, Ilkka Julkunen

**Affiliations:** 1 Virology Unit, Department of Infectious Disease Surveillance and Control, National Institute for Health and Welfare (THL), Mannerheimintie 166, FIN-00300, Helsinki, Finland; 2 Signaling Systems Laboratory, Department of Chemistry and Biochemistry, University of California, San Diego, La Jolla, CA, 92093, USA; 3 Functional Organization of the Nucleolus, RNA Biology-FRE 3402 CNRS, Université Pierre et Marie Curie, 75252, Paris cedex 5, France; 4 Nuclei and Cell Cycle, Institut Jacques Monod-UMR 7592 CNRS, Université Paris Diderot, 75205, Paris cedex 13, France

**Keywords:** Influenza A virus, NS1 protein, NoLS, Nucleolus, Nucleolin, B23, Fibrillarin

## Abstract

**Background:**

Influenza A virus non-structural protein 1 (NS1) is a virulence factor, which is targeted into the cell cytoplasm, nucleus and nucleolus. NS1 is a multi-functional protein that inhibits host cell pre-mRNA processing and counteracts host cell antiviral responses. Previously, we have shown that the NS1 protein of the H3N2 subtype influenza viruses possesses a C-terminal nuclear localization signal (NLS) that also functions as a nucleolar localization signal (NoLS) and targets the protein into the nucleolus.

**Results:**

Here, we show that the NS1 protein of the human H3N2 virus subtype interacts *in vitro* primarily via its C-terminal NLS2/NoLS and to a minor extent via its N-terminal NLS1 with the nucleolar proteins, nucleolin and fibrillarin. Using chimeric green fluorescence protein (GFP)-NS1 fusion constructs, we show that the nucleolar retention of the NS1 protein is determined by its C-terminal NLS2/NoLS *in vivo*. Confocal laser microscopy analysis shows that the NS1 protein colocalizes with nucleolin in nucleoplasm and nucleolus and with B23 and fibrillarin in the nucleolus of influenza A/Udorn/72 virus-infected A549 cells. Since some viral proteins contain NoLSs, it is likely that viruses have evolved specific nucleolar functions.

**Conclusion:**

NS1 protein of the human H3N2 virus interacts primarily via the C-terminal NLS2/NoLS and to a minor extent via the N-terminal NLS1 with the main nucleolar proteins, nucleolin, B23 and fibrillarin.

## Background

Influenza A virus genome consists of eight separate RNA segments, which encode for 12 viral structural and nonstructural proteins [1]. In addition to the viral hemagglutinin (HA), nonstructural protein 1 (NS1) is one of the major viral virulence factors. The evolution of NS genes appears to be species-specific, and the evolution of present seasonal human NS genes began in 1918, when H1N1 type viruses emerged and became pandemic [2]. With the most recent pandemic in 2009 caused by the swine-origin influenza A virus, the NS gene was changed, and it was originating from classical swine influenza viruses [3].

Influenza A virus NS1 is a multi-functional protein that contains an N-terminal dsRNA-binding domain and a C-terminal effector domain. Experiments with the human H3N2 influenza A/Udorn/72 virus demonstrated that the primary role of the NS1 dsRNA-binding activity is to inhibit the activation of IFN-induced 2’-5’ oligo (A) synthetase/RNase L pathway [4]. Instead, experiments with the mouse-adapted H1N1 influenza A/PR8/34 virus indicated that the RNA-binding domain participates in the NS1 protein-mediated inhibition of the activation of the retinoic acid-inducible gene I (RIG-I) [5,6] which is required for the influenza A virus-induced cytokine gene expression [7-9]. The effector domain of NS1 binds to two cellular proteins that are essential for the 3’ end processing of cellular pre-mRNAs [10-12]. As a result, the processing of cellular pre-mRNAs, including interferon-β (IFN-β), is inhibited [12-15]. In addition, the NS1 protein of influenza A virus has been shown to interact with protein kinase R (PKR), PACT, Crk/CrkL, TRIM25 and the p85 component of the PI-3 kinase pathway [16-21].

Eukaryotes have a nucleolus, which is a relatively large, dynamic and highly organized non-membranous subcompartment of the nucleus. The nucleolus is the site for ribosomal RNA synthesis, processing and maturation [22,23]. Recently, it has become apparent that nucleolus also has a role in regulating the cell cycle, tumor suppression and oncogenic activities, the assembly of signal recognition particle (SRP), the control of aging and the modulation of telomerase functions [24-26]. Some of these functions are mediated through the sequestration of transcription factors that control the cell cycle [26,27]. Recent studies show that the nucleolus is made up of numerous protein-protein and protein-nucleic acid interactions that are constantly changing in response to the metabolic conditions of the cell [28]. Nucleolus is divided into three discrete regions: fibrillar centres, surrounded by dense fibrillar component (DFC), which in turn is surrounded by a granular component (GC) (reviewed in [23,29]).

Three distinct subsets of nucleolar proteins have been identified. Primarily, fibrillarin is a small nucleolar ribonucleoprotein (snoRNP), which is associated with early pre-rRNA transcripts during elongation and is localizing to DFC. B23/NPM protein is a putative ribosome assembly factor involved in 28S rRNA processing and ribosome assembly that localized in the GC, while the third major nucleolar protein, nucleolin, is involved in the processing of precursor rRNAs [30].

Nucleolar localization signals (NoLSs) that regulate nucleolar localization and retention are usually rich in arginine and lysine residues, which overlap with NLSs. However, there is no obvious consensus NoLS sequence or structure [31-33]. Nuclear proteins pass through the nucleolus randomly, and those, with affinity to constitutive nucleolar components, are retained. It has been suggested that NoLSs act as retention signals rather than as classical targeting or transport signals [27,34]. It was possible to target the green fluorescent protein (GFP) into the GC of the nucleoli using sequences interacting with the acidic domains of B23/NPM [35].

Within the last few years, increasing evidence has revealed that viruses require the nucleus and in particular the nucleolus for their replication [36]. In addition to N-terminal NLS1, the C-terminus of the NS1 proteins of H3N2 and H2N2 influenza viruses have another NLS, NLS2, which also functions as a NoLS and targets the protein into the nucleolus [37]. A functional NLS2/NoLS of the Udorn H3N2 virus NS1 protein required basic arginine/lysine residues at its C-terminal end for nucleolar localization to take place [37]. Seasonal H1N1 virus NS1 proteins seem to lack these C-terminal basic residues. In addition, the C-terminal end of the H5N1 (avian virus) and H1N1pdm09 NS1 protein resembles those of the seasonal H1N1 subtype viruses or have a 15 amino acid truncation, respectively, and are thus likely to lack the C-terminal NLS2/NoLS. Murayama and co-workers identified nucleolin, an abundant nucleolar protein as a novel NS1-binding protein [38]. By laser confocal microscopy, they observed the co-localization of NS1 with nucleolin most clearly in the nucleoli, suggesting that NS1 was interacting with nucleolin during the infection [38].

Here, we show that the NS1 protein of the human H3N2 virus interacts primarily via the C-terminal NLS2/NoLS and to a minor extent via the N-terminal NLS1 with the main nucleolar proteins, nucleolin, B23 and fibrillarin. Direct interactions were observed for nucleolin and fibrillarin but not with *in vitro*-translated B23. Using GFP-NS1 fusion proteins, we show that the nucleolar retention of the NS1 protein is determined by its C-terminal NLS2/NoLS *in vivo*. Confocal laser microscopy results show that the NS1 protein colocalizes with nucleolin in nucleolus and nucleoplasm and with B23 and fibrillarin in nucleolus in influenza A/Udorn/72 virus-infected A549 cells.

## Results

### NS1 proteins, containing a C-terminal NLS2/NoLS, are targeted into the nucleoli in H3N2 subtype influenza A virus-infected A549 cells

**Figure 1:**
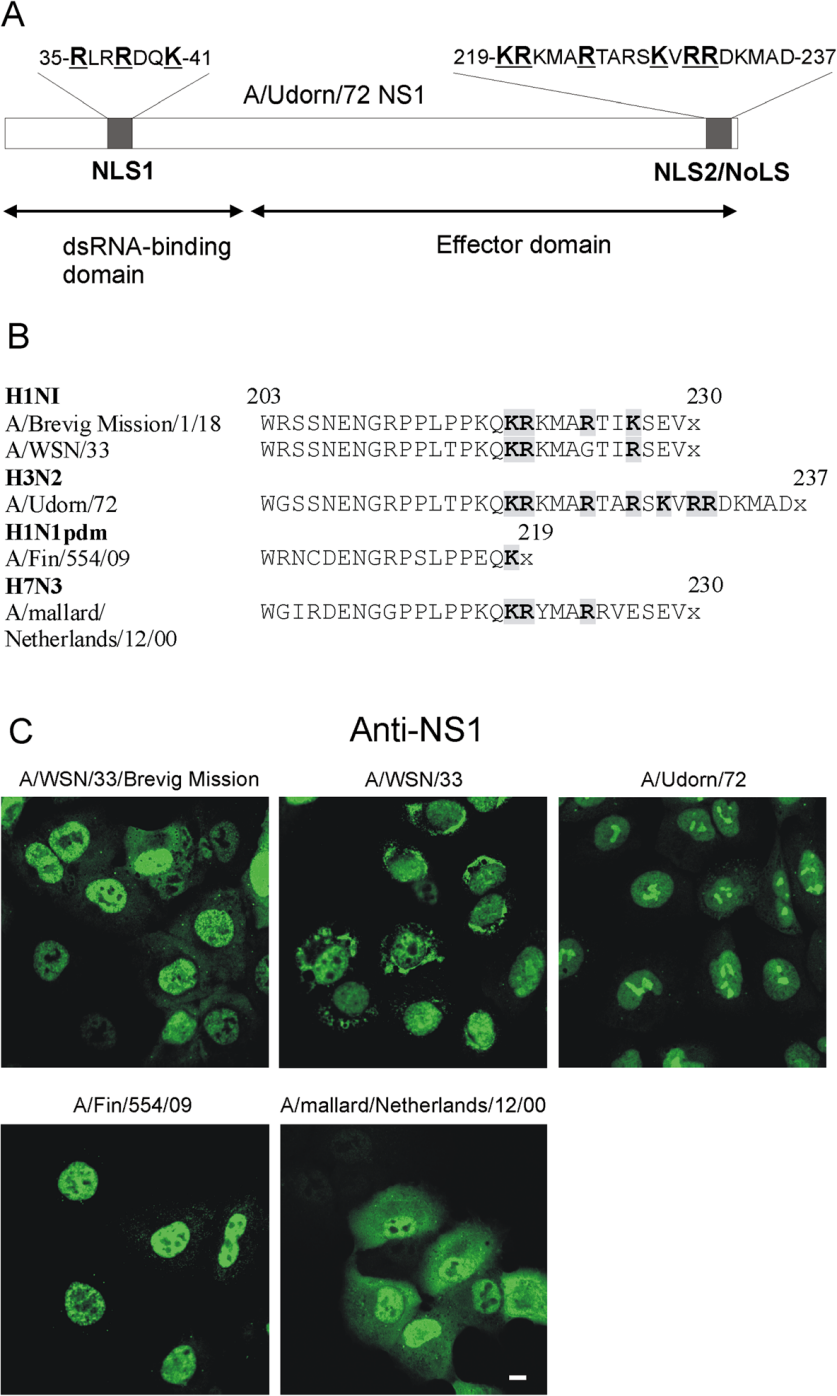
**NS1 protein in H3N2 type influenza A viruses targets into the nucleolus in virus-infected A549 cells.** (**A**) A schematic representation of the intracellular targeting signals of the A/Udorn/72 virus NS1 protein. (**B**) Variations of putative C-terminal NLS2/NoLSs in selected influenza A virus NS1 proteins are presented. Critical amino acids 219, 220, 224, 227, 229, 231, and 232 that are involved in targeting the NS1 protein into the nucleus and nucleolus [37] are shown in boldface. (**C**) A549 cells grown directly on coverslips were infected with recombinant influenza A/WSN/33 (A/Brevig Mission/1/18 segm 8) or wt A/WSN/33 (H1N1), A/Udorn/72 (H3N2), A/Fin/554/09 (H1N1pdm09) and A/Mallard/Netherlands/12/00 (H7N3) viruses (MOI 5) for 9 h as indicated in the figure. After fixation, the cells were stained with rabbit anti-NS1 and fluorescein isothiocyanate-labeled anti-rabbit immunoglobulins, followed by analysis with confocal laser microscopy. Bar, 5 μm.

We have previously shown that the NS1 protein of the H3N2 subtype influenza A viruses contain a C-terminal NLS2/NoLS (Figure 1A) that targets the protein into the nucleoli in virus-infected cells [37]. To further confirm this observation, we infected A549 cells with wild type recombinant H1N1 subtype A/WSN/33 and A/WSN/33 virus expressing the 1918 NS gene (A/Brevig Mission/1/18 segm 8), H3N2 subtype A/Udorn/72, H1N1pdm09 subtype A/Fin/554/09 and avian H7N3 subtype A/mallard/Netherlands/12/00 viruses for nine h. After fixation of the cells, NS1 protein expression was detected using anti-NS1 protein-specific antibodies (Figure 1C). In general, all analyzed NS1 proteins were mainly observed to be localized in the cell nuclei. As an exception, the NS1 protein of the A/Udorn/72 virus was observed in the cell nucleus and enriched in nucleoli while a weaker nucleolar staining was seen in A/WSN/33 virus-infected cells (Figure 1C). The NS1 proteins of the recombinant A/WSN/33 (A/Brevig Mission/1/18 segm 8), A/mallard/Netherlands/12/00 and H1N1pdm09 A/Fin/554/09 viruses were mainly excluded from nucleoli, even though clear nucleolar staining was still detected in few cells.

NS1 protein can change its location over the time course of infection. NS1 is nuclear in the beginning and both nuclear and cytoplasmic at later stages of the infection [37]. Cytoplasmic translocation is mediated by a nuclear export signal (NES) [39]. Cellular localization emphasizes the specific functions of the NS1 protein in the cytoplasm, nucleus and nucleolus.

### The chimeric GFP-NS1 protein that contains the C-terminus of the H3N2 subtype influenza A/Udorn/72 virus is targeted into the nucleoli in transiently transfected HuH7 cells

**Figure 2:**
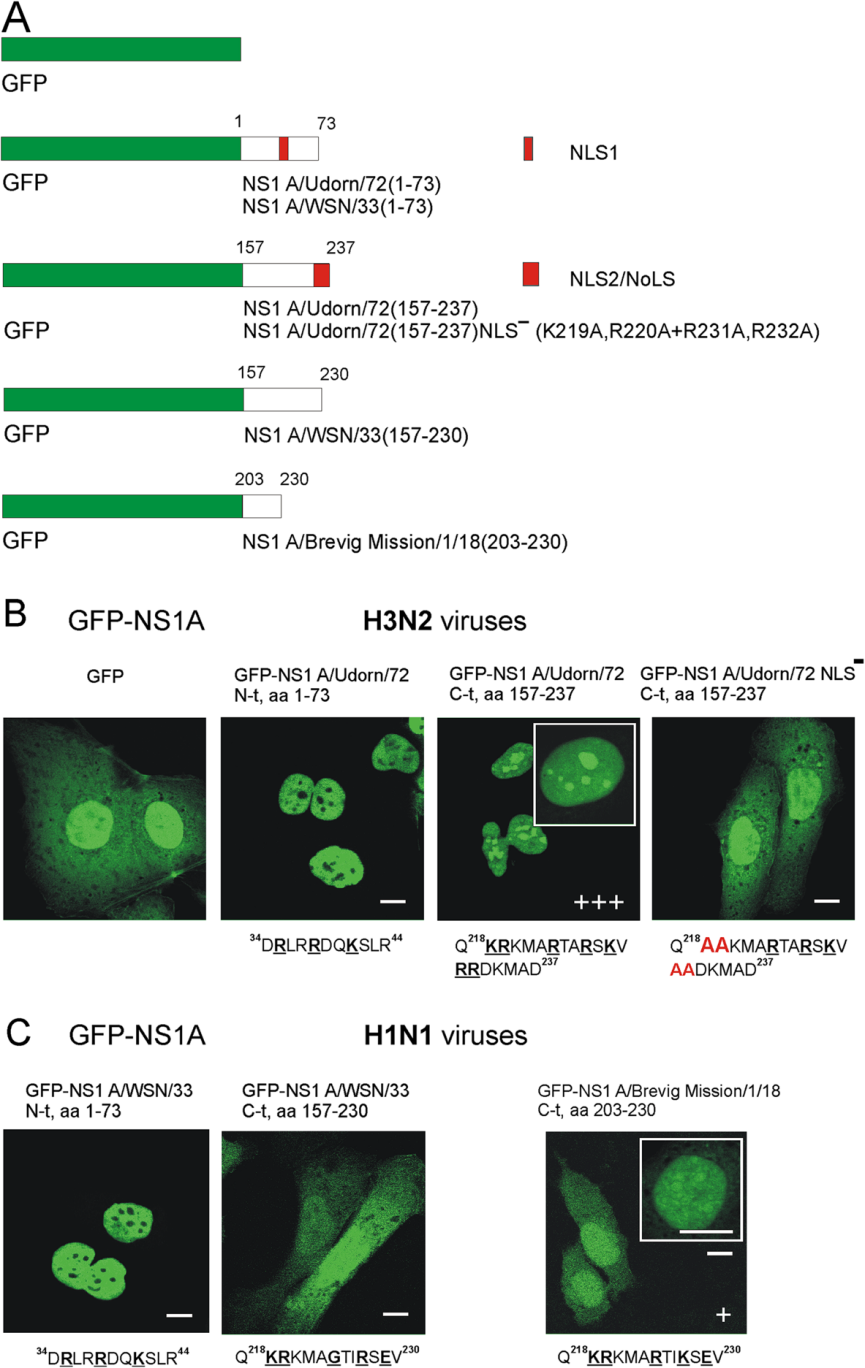
**The C-terminal end of the NS1 protein in H3N2 type influenza A viruses encode for a functional NoLS.** (**A**) The N- and C-terminal fragments of the NS1 genes, encoding for amino acids 1–73 and 157–237 in A/Udorn/72, amino acids 1–73 and 157–230 in A/WSN/33 and amino acids 203–230 in A/Brevig Mission/1/18, were inserted into the GFP expression vector pCMX-SAH/Y145F to express GFP-NS1 fusion proteins. Mutations in the C-terminal NLS2/NoLS of A/Udorn/72 virus NS1 protein were performed as described in Materials and Methods. Red boxes indicate the positions of N-terminal NLS1 and C-terminal NLS2/NoLS in NS1 protein. (**B**) HuH7 cells were transiently transfected with GFP, GFP-NS1 fusion and GFP-NS1 fusion mutant (K219A,R220A + R231A,R232A) A/Udorn/72 gene constructs, as indicated in the figure, for 48 h. The intensity of nucleolar localization was scored by immunofluorescence microscopy as no nucleolar staining (−) or weak (+), moderate (++) or strong (+++) nucleolar staining. Critical basic amino acids involved in nuclear/nucleolar targeting are marked in boldface and underlined. Mutated amino acids (K219A,R220A + R231A,R232A) are marked in red. (**C**) HuH7 cells were transiently transfected with GFP and the GFP-deletion gene constructs of NS1 A/WSN/33 and NS1 A/Brevig Mission/1/18 for 48 h as indicated in the figure. Critical and mutated amino acids are marked as above. Bars, 5 μm.

Next, fragments of the NS1 genes encoding for amino acids 1–73 and 157–237 of the NS1 protein of A/Udorn/72 virus, for amino acids 1–73 and 157–230 of the NS1 protein of A/WSN/33 virus and for amino acids 203–230 of the NS1 protein of A/Brevig Mission/1/18 virus were inserted into a GFP expression vector pCMX-SAH/Y145F and GFP-NS1 fragment fusion proteins were transiently expressed in HuH7 cells (Figure 2). The N-termini (amino acids 1–73) of the H3N2 and H1N1 subtype influenza virus NS1 proteins, containing the NLS1 (Figure 2A), efficiently targeted the fusion proteins into the cell nucleus but not to the nucleoli which appeared to remain negative (Figure 2B and C). The C-terminal part (amino acids 157–237) of the NS1 protein of the H3N2 subtype influenza virus, containing the NLS2/NoLS, also targeted the fusion protein into the cell nucleus but, in addition, concentrated strongly in the nucleoli (Figure 2B). The mutation of several basic arginine (R) and lysine (K) residues to alanine (A) of the NLS2/NoLS (K219A,R220A + R231A,R232A) abolished the accumulation of this fusion protein into the host cell nucleus and nucleoli and led to a localization of the fusion protein similar to that of GFP (Figure 2B). Similarly, the GFP-NS1 A/WSN/33 (amino acids 157–230) and GFP-NS1 A/Brevig Mission/1/18 (amino acids 203–230) fusion proteins did not clearly accumulate neither in the nucleus nor in nucleoli and exhibited a localization rather comparable to that of the GFP alone (Figure 2C).

### The C-terminal NoLS of the influenza A/Udorn/72 NS1 protein binds to nucleolar proteins nucleolin, B23 and fibrillarin

**Figure 3:**
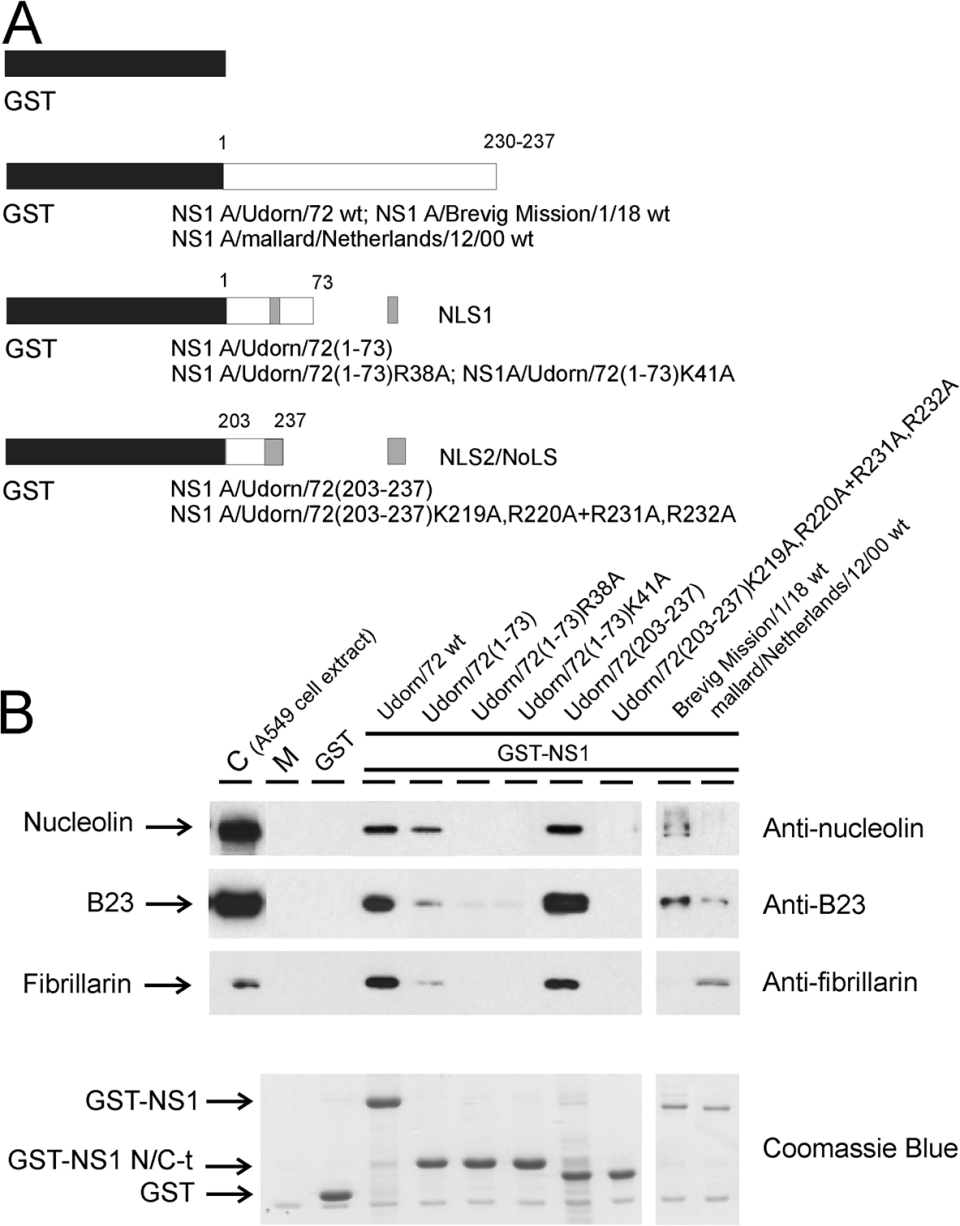
**The C-terminal NoLS of the NS1 protein in H3N2 subtype influenza A viruses binds nucleolar nucleolin, B23 and fibrillarin in a GST-NS1 pull-down experiment.** (**A**) N- and C-terminal fragments of the NS1 gene encoding for amino acids 1–73 and 203–237 in A/Udorn/72 and wt genes, encoding for amino acids 1–237 in A/Udorn/72 and amino acids 1–230 in A/Brevig Mission/1/18 and in A/Mallard/Netherlands/12/00, were inserted into the GST expression vector pGEX 2 T(+) to express GST-NS1 fusion proteins. Mutations were created into the N-terminal NLS1 and C-terminal NLS2/NoLS in the A/Udorn/72 virus NS1 protein are as indicated in the figure. (**B**) The cell extracts of cultured A549 cells were prepared, and proteins in cell extracts were allowed to bind to *E. coli*-expressed and Sepharose-immobilized GST, GST-NS1 A/Udorn/72 wt, GST-NS1 A/Udorn/72(1–73), GST-NS1 A/Udorn/72(1–73)R38A, GST-NS1 A/Udorn/72(1–73)K41A, GST-NS1 A/Udorn/72(203–237), GST-NS1 A/Udorn/72(203–237)K219A,R220A + R231A,R232A, GST-NS1 A/Brevig mission/1/18 wt and GST-NS1 mallard/Netherlands/12/00 wt at +4 °C for 1 h. Sepharose-bound proteins were dissolved in Laemmli sample buffer and separated on 8 % SDS-PAGE. Western blots were stained with anti-nucleolin, -B23 and -fibrillarin immunoglobulins. As a control, A549 cell extract, containing 10 μg of proteins, was stained in lane C. M indicates the Sepharose matrix-bound proteins. A similar gel was also stained with Coomassie Brilliant Blue to visualize the amount of Sepharose-immobilized GST and GST-NS1 fusion proteins.

Next, we carried out pull-down experiments using A549 cell extracts to identify the nucleolar proteins that could interact with influenza A virus NS1 protein. In these experiments wt NS1 protein of the H3N2 subtype A/Udorn/72 influenza virus was observed to interact with nucleolin, B23 and fibrillarin (Figure 3B). The 1918 H1N1 subtype A/Brevig Mission/1/18 virus NS1 protein was observed to interact with nucleolin and B23 and the H7N3 subtype A/mallard/Neatherlands/12/00 virus NS1 protein with B23 and fibrillarin but these interactions were weak (Figure 3B). Unfortunately, the expression levels of GST-NS1 A/Brevig Mission/1/18 and GST-NS1 A/mallard/Neatherlands/12/00 fusion proteins were much lower than that of GST-NS1 A/Udorn/72. Thus, the relative binding intensities of these proteins with nucleolar proteins could not be compared (Figure 3B). Previously, it has been suggested that the N-terminal RNA-binding domain of the NS1 binds nucleolin [38]. To further confirm this observation in our experimental system, we used GST-NS1 A/Udorn/72 (amino acids 1–73) fusion protein (Figure 3A) in pull-down experiments and found a weak binding of nucleolin, B23 and fibrillarin to the N-terminal part of NS1. The binding was totally abolished by R38A and K41A substitution mutations to the RNA-binding domain, which are the critical amino acids that form the NLS1 of the NS1 protein (Figure 3B). Conversely, the GST-NS1 A/Udorn/72 (amino acids 203–237) fusion protein pulled-down the nucleolar proteins, nucleolin, B23 and fibrillarin at least as well as the wt NS1 protein did (Figure 3B) showing the predominant role of the C-terminus of the NS1 protein in the interactions of NS1 with nucleolin, B23 and fibrillarin. These bindings were totally abolished by K219A,R220A + R231A,R232A substitution mutations (Figure 3B) highlighting the importance of NLS2/NoLS sequences in the interaction with nucleolar proteins.

### The C-terminal NLS2/NoLS of the NS1 protein of the influenza A/Udorn/72 virus bound *in vitro*-translated nucleolin and fibrillarin but not *in vitro*-translated B23 in a GST pull-down experiment

**Figure 4:**
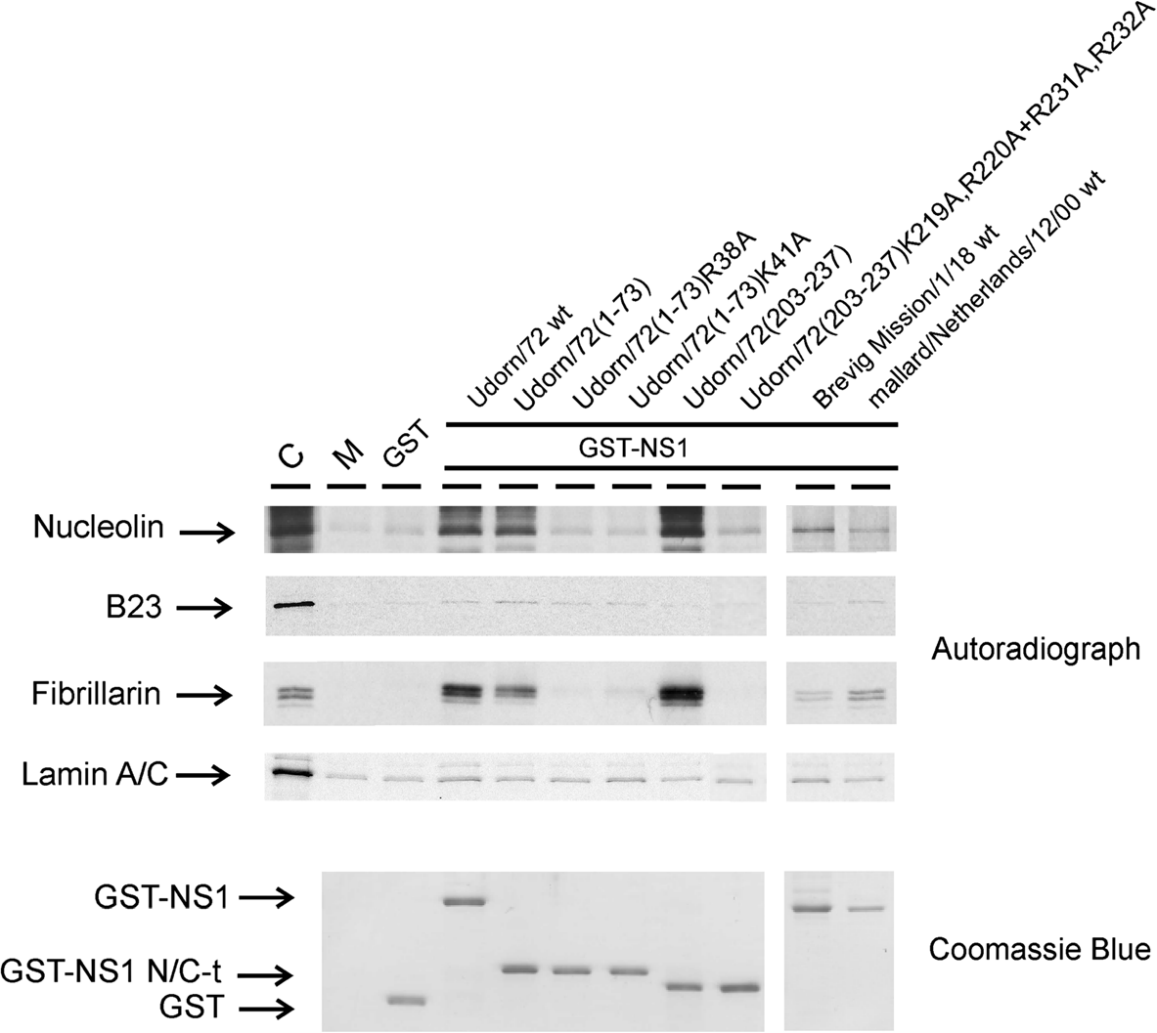
**The C-terminal NoLS of the NS1 protein in the H3N2 subtype influenza A viruses binds *in vitro*-translated nucleolar nucleolin and fibrillarin in a GST pull-down experiment.** [^35^S]-labeled and *in vitro*-translated nucleolin, B23, fibrillarin and, as a control, lamin A/C were allowed to bind to *E. coli*-expressed and Sepharose-immobilized GST, GST-NS1 A/Udorn/72 wt, GST-NS1 A/Udorn/72(1–73), GST-NS1 A/Udorn/72(1–73)R38A, GST-NS1 A/Udorn/72(1–73)K41A, GST-NS1 A/Udorn/72(203–237), GST-NS1 A/Udorn/72 (203–237)K219A,R220A + R231A,R232A, GST-NS1 A/Brevig mission/1/18 wt and GST-NS1 A/mallard/Netherlands/12/00 wt at +4 °C for 1 h. Sepharose-bound proteins were dissolved in Laemmli sample buffer, separated on 8 % SDS-PAGE and autoradiographed. Input (lane C) was 1:10 of the amount of [^35^S]-labeled protein that was used in the binding experiment. M indicates the Sepharose matrix-bound proteins. A similar gel was also stained with Coomassie Blue to visualize the amount of Sepharose-immobilized GST and GST-NS1 fusion proteins.

Next, to verify our previous binding results and to know whether the interactions between NS1 protein and nucleolar proteins occur directly or indirectly via other proteins, we carried out pull-down experiments using *in vitro-*translated ^35^S]-methionine-labeled nucleolin, B23 and fibrillarin. The procedure is described in the legend of Figure 4 and the GST-NS1 fusions were the same as in the experiments described above (Figure 3). The wt NS1 protein of the H3N2 subtype A/Udorn/72 influenza virus bound directly the in *vitro-*translated nucleolin and fibrillarin but not the *in vitro-*translated B23 (Figure 4). Instead, the wt NS1 protein of the H1N1 subtype A/Brevig Mission/1/18 virus bound only weakly nucleolin and fibrillarin, while the wt NS1 protein of the H7N3 subtype mallard/Neatherlands/12/00 virus showed a weak binding to fibrillarin but not to the other tested nucleolar proteins (Figure 4). The GST-NS1/Udorn/72 (amino acids 1–73) fusion protein clearly pulled-down nucleolin and fibrillarin showing the role of the N-terminus of the NS1 protein of the H3N2 subtype A/Udorn/72 influenza virus in these interactions. The interactions were totally abolished by R38A and K41A substitution mutations (Figure 4) and therefore the interactions are most likely dependent on NLS1 of the NS1 protein. The GST-NS1 A/Udorn/72 (amino acids 203–237) fusion protein bound nucleolin and fibrillarin as efficiently as the wt NS1 protein (Figure 4) showing the crucial role played by the C-terminus of the NS1 protein in these direct interactions. This binding was totally abolished by K219A,R220A + R231A,R232A substitution mutations (Figure 4) of the previously identified NLS2/NoLS of the Udorn virus NS1 protein [37]. We can therefore conclude that the NLS2/NoLS is implicated in direct interactions between the NS1 protein with nucleolin and fibrillarin.

### The NS1 protein colocalized with nucleolin and to a minor extent with B23 and fibrillarin in influenza A/Udorn/72 virus-infected A549 cells

**Figure 5:**
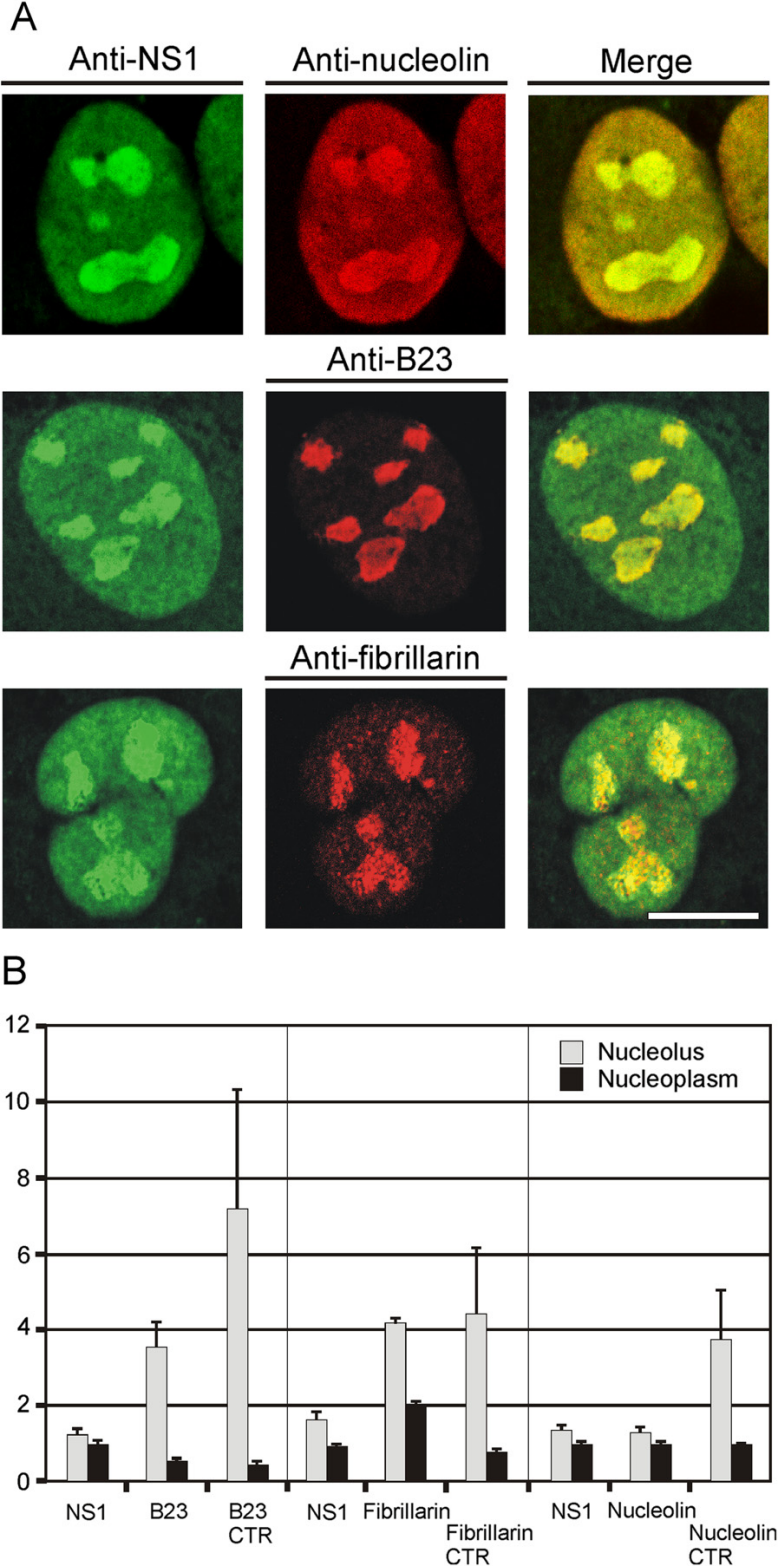
**NS1 protein of the H3N2 subtype influenza A virus colocalizes with nucleolin in the nucleolus and nucleoplasm and with B23 and fibrillarin in the nucleolus in influenza A/Udorn/72 virus-infected A549 cells.** (**A**) A549 cells were infected with influenza A/Udorn/72 wt viruses (MOI 5) for eight hours, fixed with 3 % paraformaldehyde and stained with rabbit or guinea pig anti-NS1 immunoglobulins, followed by fluorescein isothiocyanate-labeled goat anti-rabbit or anti-guinea pig immunoglobulins. To detect protein co-localization, cells were double-stained with mouse anti-nucleolin and anti-B23 or rabbit anti-fibrillarin immunoglobulins, followed by Rhodamine Red-X-labeled goat anti-mouse or anti-rabbit immunoglobulins, respectively. The colocalization of proteins was visualized with confocal laser microscopy. Only the nuclei of the cells are presented. Bar, 5 μm. (**B**) The relative distribution of both viral and nucleolar proteins in the nuclei, as illustrated in A, is presented. The relative intensity of fluorescence in the nucleolus and nucleoplasm was quantified by ImageJ software (mean of 30 cells in each case). The quantification was also performed on 30 control (CTR) cells not expressing NS1 for each nucleolar protein.

Next, we analyzed the colocalization of H3N2 subtype influenza A virus NS1 protein with nucleolin, B23 and fibrillarin (Figure 5). The procedure is described in the legend of Figure 5. The strongest colocalization was observed between NS1 and nucleolin as evidenced by the merge of green and red signals visible in yellow in nucleoplasm and nucleolus. On the contrary, the colocalization of the NS1 protein with fibrillarin and B23 was mainly observed in the nucleolus (yellow in the merge images). The next step was the quantification of the relative distribution of both viral and nucleolar proteins in the nuclei. The signals were measured using ImageJ software in 3 groups of 30 cells, in which GFP-NS1 is expressed and either B23, or fibrillarin or nucleolin was detected by antibodies. The quantification was also performed on 30 control (CTR) cells not expressing NS1 for each nucleolar proteins. The fluorescence intensity was measured in the nucleoplasm and nucleoli of each cell (green and red fluorescence for cells expressing NS1 and red fluorescence for control cells) and was normalized to the mean nuclear fluorescence value to compare the concentration of B23, nucleolin and fibrillarin respectively very abundant, abundant and less abundant nucleolar proteins and the distribution of NS1 in each group. The quantification indicated that the amount and distribution of NS1 is quite similar in the NS1-B23, NS1-fibrillarin, or NS1-nucleolin cells (Figure 5B) indicating that the expression of the NS1 protein is comparable in each group. The relative concentration of B23 in nucleoli in the presence of NS1 is high compared to the nucleoplasm but only half of that of the control cells (Figure 5B) suggesting a reduced relative concentration of B23 in nucleoli in the presence of NS1. The relative concentration of fibrillarin in the presence of NS1 is higher in nucleoli than in nucleoplasm but its relative concentration in the nucleoplasm is two-fold higher as compared to the control cells (Figure 5B). This could be due to the modification of the nuclear traffic of fibrillarin. The same relative distribution was observed for nucleolin and NS1 (Figure 5B) that is in accordance with our demonstration of the interaction between these proteins (Figures 3 and 4). It is possible that this interaction modifies the traffic of nucleolin in the cell because the relative nucleolar amount is higher in the control cells than in presence of NS1 (Figure 5B).

To analyze the relative re-distribution of the nucleolar proteins in the presence of NS1, the relative intensities of nucleolus/nucleoplasm ratios for both NS1 and nucleolar proteins was compared in each cell. The ratio for NS1/B23 is 0.19, for NS1/fibrillarin it is 0.77 and for NS1/nucleolin it is 1.02 (Table 1). The ratios clearly demonstrate that the relative distribution of nucleolin and NS1 are similar in nucleolus and nucleoplasm in each cells (ratio 1) in contrast to fibrillarin and B23 (ratios 0.77 and 0.19 respectively), which show relatively higher amounts in the nucleoli than NS1. This analysis of the relative distribution of NS1 and the nucleolar proteins indicates a differential effect probably related to their function. It is interesting to note that fibrillarin is involved at the early stage of rRNA processing, B23 at late stage and nucleolin at different stages of rRNA processing.

**Table 1:** The relative re-distribution of nucleolar proteins in the presence of NS1

**Ratio**	**Ratio**	**Ratio**
**NU/NCP**	**NU/NCP**	**NS1/Nucleolar protein**
NS1	B23	
**1.36**	**8.89**	**0.19**
NS1	Fibrillarin	
**1.80**	**2.10**	**0.77**
NS1	Nucleolin	
**1.44**	**1.43**	**1.02**

The relative intensities of nucleolus/nucleoli ratios for both NS1 and nucleolar proteins is compared in each cell. Quantification was done by ImageJ software (mean of 30 cells in each case). NU: nucleolus; NCP: nucleoplasm.

### High expression level of the HIV-1 Rev protein inhibited the nucleolar targeting of the H3N2 subtype influenza A virus GFP-NS1 protein fusion (amino acids 203–237)

**Figure 6:**
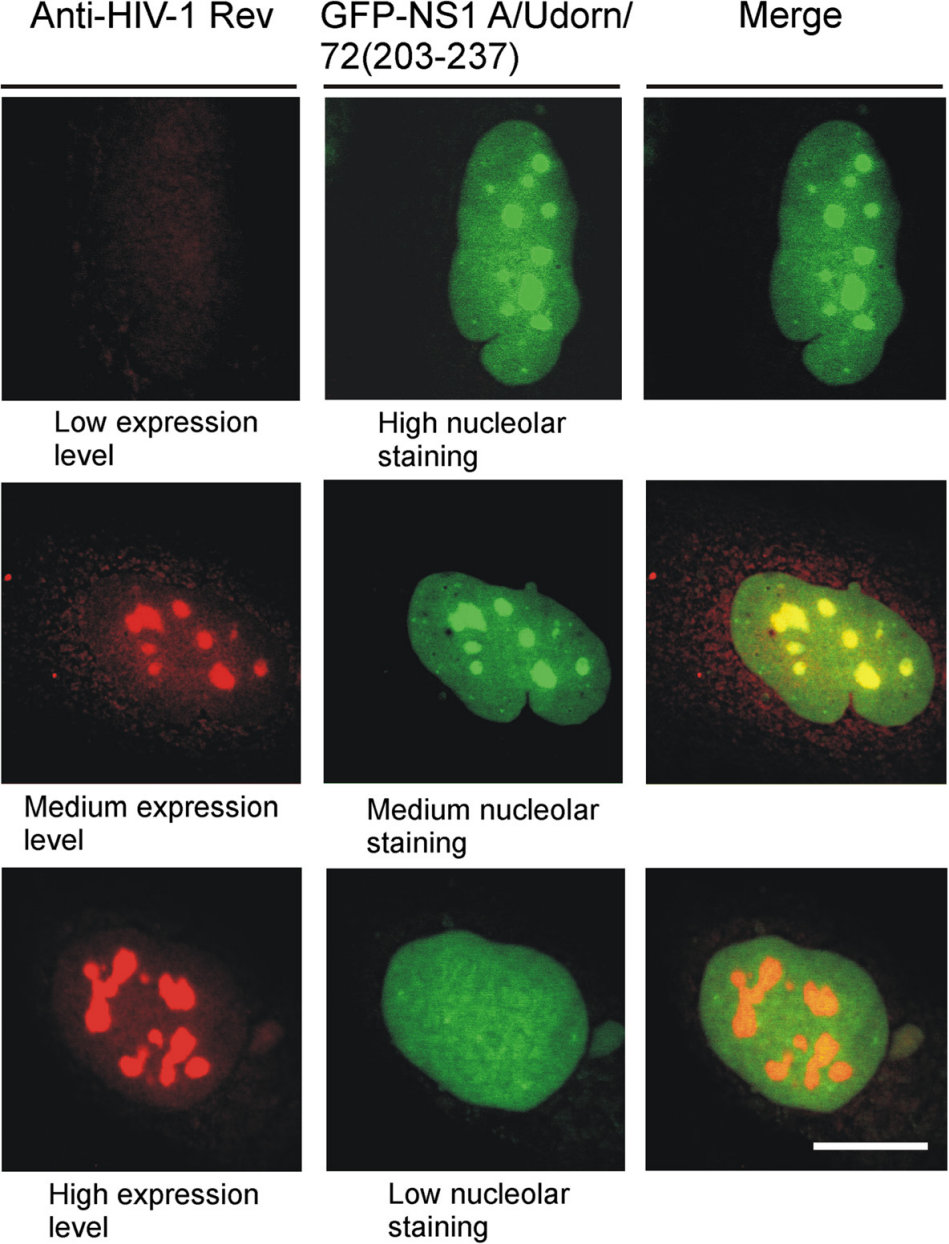
**Chimeric GFP-NS1 A/Udorn/72/(203–237) colocalizes with HIV-1 Rev protein in the nucleolus, and a high expression level of HIV-1 Rev protein totally displaced the GFP-NS1(203–237) fusion protein from the nucleoli to the nucleus.** HuH7 cells were grown on coverslips for 24 h and then transiently transfected with HIV-1 Rev and chimeric GFP-NS1 A/Udorn/72/(203–237) gene constructs for 48 h. After fixation, the cells were stained with rabbit anti-HIV-1 Rev and Rhodamine Red-X -labeled goat anti-rabbit immunoglobulins, followed by analysis with confocal laser microscopy. A high expression level of HIV-1 Rev protein totally displaced the GFP-NS1(203–237) fusion protein from the nucleoli to the nucleus (bottom panels). Cells, expressing low, medium or high amounts of nucleolar HIV-1 Rev protein, were selected. Only the nuclei of the cells are presented. Bar, 5 μm.

Previously, it has been shown that HIV-1 Rev protein targets into the nucleolus [40] and binds to nucleolar B23 [41]. In order to further characterize NS1 nucleolar binding structures, we coexpressed the GFP-NS1 A/Udorn/72 (amino acids 203–237) fusion protein and the HIV-1 Rev protein and analyzed their possible nucleolar colocalization. The procedure is described in the legend of Figure 6. In this experiment, cells, expressing low, medium or high amounts of nucleolar HIV-1 Rev protein, were selected by the relative intensity of fluorescent light. 30 cells in all three groups were examined. As shown in Figure 6, the GFP-NS1( 203–237) fusion protein and HIV-1 Rev protein completely colocalized in the nucleolus. Interestingly, a high expression level of HIV-1 Rev protein totally displaced the GFP-NS1 protein fusion from the nucleoli to the nucleus. The phenomenon was clearly seen in all cells expressing high level of HIV-1 Rev protein. This further indicates that NS1 protein, and specifically its C-terminal NLS2/NoLS domain, has a strong nucleolar retention signal that is likely binding to similar nucleolar proteins/structures as HIV-1 Rev protein does.

## Discussion

Many different viruses target the nucleolus to disrupt host-cell functions and to recruit cellular proteins to aid in virus replication. As the primary site of the replication of positive-strand RNA viruses and most negative-strand RNA viruses is the cytoplasm, the reason for RNA viruses to target nuclear structures is not immediately obvious. However, among RNA viruses, influenza virus is an exception, since all the essential viral replication events take place in the nucleus.

We have previously shown that the NS1 protein of the H3N2 subtype influenza A virus contains a C-terminal NLS2/NoLS that efficiently targets the protein into the nucleoli in virus-infected cells [37]. Murayama and co-workers identified the nucleolar protein, nucleolin, as a novel NS1-binding protein [38]. They also suggested that the N-terminal part of the NS1 protein was responsible for this binding, as judged by a GST pull-down assay with the GST-fused functional domains of NS1. By laser confocal microscopy, they observed that NS1 colocalized with nucleolin most clearly in the nucleoli, indicating that NS1 was interacting with nucleolin during the infection [38].

In the present study, instead, we show that the NS1 protein of the human H3N2 subtype influenza A virus can interact, primarily via its C-terminal NoLS, with the main nucleolar proteins, nucleolin, B23 and fibrillarin *in vitro*. Using GFP-NS1 fusion proteins, we observed that the N-terminal part of both the H3N2 and H1N1 subtype influenza NS1 protein, containing a functional NLS1, targeted the chimeric proteins into the cell nucleus but not to the nucleolus (Figure 2B and C). However, only the C-terminus of the NS1 protein of the H3N2 subtype A influenza virus, containing a functional NLS2/NoLS, targeted the chimeric protein into the cell nucleus and nucleoli (Figure 2B and C). Mutations in the NLS2/NoLS abolished both the nuclear and nucleolar targeting (Figure 2B). A chimeric GFP-NS1 A/Brevig Mission/1/18(203–230) protein was only faintly targeted into the cell nucleus and nucleolus (Figure 2C). These results are in agreement with our previous results showing that all types of influenza A virus NS1 proteins have an N-terminal NLS1, while H3N2 subtype viruses also have an additional NLS, a C-terminal NLS2, which also functions as a NoLS [37].

As mentioned above, Murayama and co-workers showed that the N-terminal RNA-binding domain of the NS1 protein was responsible for the binding to nucleolin [38]. Using GST-NS1 A/Udorn/72(1–73) fusion protein construct and A549 cell extracts, we were also able to see a weak binding of nucleolin, B23 and fibrillarin to the N-terminal part of a chimeric NS1 protein (Figure 3). The binding was abolished by R38A and K41A substitution mutations to the RNA-binding domain of the NS1 protein (Figure 3B). The same amino acids are also critical for the importin α-binding and nuclear translocation of NS1 protein via the nuclear transport machinery [37]. Instead, the C-terminal part of the NS1 protein in the chimeric construct GST-NS1/A/Udorn/72(203–237) bound nucleolin, B23 and fibrillarin as well as the wt NS1 protein did (Figure 3B). This binding was totally abolished by K219A,R220A + R231A,R232A substitution mutations (Figure 3B), indicating the NLS2/NoLS to be primarily responsible for the binding of NS1 to nucleolar proteins.

The signals that govern the nucleolar localization and retention of different proteins are not well defined. The motifs involved are usually rich in arginine and lysine residues, however, there is no obvious consensus sequence or structure. Proteins that localize in the nucleolus can also have nuclear import and export motifs. It is unclear whether nucleolar proteins are specifically localized, targeted, accumulated or just retained in the nucleolus and consequently the sequences associated with the nucleolar localization have thus been designated as nucleolar targeting signals and/or nucleolar retention signals. In many cases, proteins that localize in the cytoplasm and in the nucleus and/or nucleoli contain multiple signals that determine their subcellular localization. This highlights the difficulty in identifying truly functional NoLSs. Moreover, proteins that localize in the nucleoli must first be imported into the nucleus and therefore they likely possess both NLS and NoLS whose sequences may overlap (Reviewed in [29].

Our previous [37] and present data clearly shows that the nucleolar retention of the H3N2 subtype influenza A virus NS1 protein is mediated primarily by its C-terminal NLS2/NoLS. However, the N-terminal NLS1 may also mediate weak nuclear retention of NS1 protein, since the H1N1pdm NS1 protein was weakly localized into the nucleoli, even if it lacked the C-terminal NLS2/NoLS due to a stop codon at position 220 (Figure 1). There may also be viral species-specific differences, since the 230-amino-acid-long NS1 of the avian A/mallard/Netherlands/12/00 virus completely failed to target into the nucleoli (Figure 1).

Nucleoli are compact structures, and thus it was quite expected that all three major nucleolar proteins co-precipitated with the H3N2 virus NS1 protein in GST pull-down experiments. However, *in vitro*-translated nucleolar proteins showed that nucleolin and fibrillarin specifically bound to the C-terminal part of the NS1 protein in a manner similar to that seen with A549 cell extracts (compare Figures 3 and 4). Instead, *in vitro*-translated B23 did not bind to the NS1 protein suggesting that this protein has no intrinsic binding ability to NS1. However, it coprecipitated with other nucleolar proteins in GST pull-down experiments (Figure 4). It is also possible that *in vitro*-translated B23 is misfolded or requires additional posttranslational modifications, such as phosphorylation for the NS1 interaction to take place.

Laser confocal microscopy showed a clear colocalization of NS1 with nucleolin, B23 and fibrillarin, indicating that NS1 was interacting with these proteins during the virus infection (Figure 5A). The HIV-1 Rev protein has previously been shown to localize into the nucleolus (reviewed in [42]). Like many viral proteins that target into the nucleolus, Rev has also been shown to contain a functional NoLS [43]. In the nucleolus, HIV-1 Rev protein binds to nucleolar B23 [41] and localizes to the combined regions of the dense fibrillar component and the granular component. To further identify the interaction partners of NS1 in the nucleolus, we analyzed nucleolar co-localization of the GFP-NS1 A/Udorn/72/(203–237) and HIV-1 Rev proteins. GFP-NS1 A/Udorn/72/(203–237) and HIV-1 Rev proteins colocalized in the nucleolus, and high expression level of the HIV-1 Rev protein totally displaced the chimeric GFP-NS1(203–237) protein from the nucleolus. This may indicate that these proteins are binding to similar structures within the nucleolus or that all nucleolar proteins have a very tight interaction with each others.

Further analysis of the role of the nuclear retention of the NS1 protein during influenza virus infection is needed. The fact that viral proteins contain NoLSs is an indication that viruses have evolved specific nucleolar functions. There is emerging evidence that the disruption of the nuclear or nucleolar trafficking by some positive-stranded RNA virus proteins may have a role in viral pathogenesis.

## Conclusions

The present study shows that the NS1 protein of the human H3N2 subtype virus interacts *in vitro* primarily via its C-terminal NLS2/NoLS and to a minor extent via its N-terminal NLS1 with the nucleolar proteins, nucleolin and fibrillarin. Using chimeric green fluorescence protein (GFP)-NS1 fusion constructs, we show that the nucleolar retention of the NS1 protein is determined by its C-terminal NLS2/NoLS *in vivo*. Confocal laser microscopy analysis shows that the NS1 protein colocalizes with nucleolin in nucleoplasm and nucleolus and with B23 and fibrillarin in the nucleolus of influenza A/Udorn/72 virus-infected A549 cells. Since some viral proteins contain NoLSs, it is likely that viruses have evolved specific nucleolar functions. It is thus likely that the nucleolar targeting function of NS1 protein plays a role in the pathogenesis of human influenza A viruses that, undoubtedly, deserves further studies.

## Methods

### Cells

Human A549 lung carcinoma cell line (ATCC, CCL 185) was maintained in a continuous culture in minimum Eagle’s medium-α (Invitrogen Corp., Carlsbad, CA, USA) supplemented with 0.6 μg/ml penicillin, 60 μg/ml streptomycin and 10% fetal calf serum (Integro, BV, Dieren, The Netherlands). Human hepatocellular carcinoma HuH7 [44] cells were maintained in minimum Eagle’s medium-α with supplements as above.

### Generation of reverse genetics A/WSN/33 (A/Brevig Mission/1/18 segm 8) virus

Reverse genetics in the A/WSN/33 H1N1 background [45] was performed using a pHH21 vector containing a wild-type synthetic gene fragment encoding the A/Brevig Mission/1/18 NS gene [17]. Viruses were propagated using Madin-Darby canine kidney (MDCK) cells [46].

### Viruses and infections

A549 cells, grown on 6-well plates or on glass coverslips on 24-well plates, were infected with the following viruses: A/Udorn/72 (H3N2), A/WSN/33 (H1N1), A/WSN/33 (A/Brevig Mission/1/18 segm 8) (H1N1), A/Fin/554/09 (H1N1pdm09) and A/mallard/Netherlands/12/2000 (H7N3). Virus stocks were cultivated in eight-day-old embryonated chicken eggs and stored at −70 °C. The hemagglutination titers of the stock viruses ranged from 64 to 256, and the infectivity of the virus stocks in A549 cells was 1-4 × 10^7^ pfu/ml. The multiplicity of infection (MOI) used in the experiments varied from 0.5 to 5 pfu/cell.

### Antibodies

In Western blot analysis mouse monoclonal anti-nucleolin (anti-C23; sc-8031; 1:1000 dilution; Santa Cruz Biotechnology, Santa Cruz, CA, USA), mouse monoclonal anti-B23 (sc-32256; 1:2000 dilution; Santa Cruz Biotechnology) and rabbit polyclonal anti-fibrillarin (#2639; 1:200 dilution; Cell Signaling Technology, Inc., Beverly, MA, USA) immunoglobulins were used. Secondary HRP-conjugated goat anti-mouse and goat anti-rabbit immunoglobulins (1:2000 dilution; Daco, Glostrup, Denmark) were used as suggested by the manufacturer. Guinea pig anti-influenza NS1 [37] and rabbit anti-HIV-1 Rev [47] immunoglobulins were used in confocal laser microscopy. Also mouse monoclonal anti-nucleolin (anti-C23; sc-8031; 1:100 dilution; Santa Cruz Biotechnology), mouse monoclonal anti-B23 (sc-32256; 1:200 dilution; Santa Cruz Biotechnology) and rabbit polyclonal anti-fibrillarin (#2639; 1:25 dilution; Cell Signaling Technology, Inc.) immunoglobulins were used. Secondary antibodies used were Rhodamine- Red-X- or FITC-labeled goat anti-guinea pig, anti-mouse or anti-rabbit immunoglobulins, respectively (1:100 dilution; Jackson ImmunoResearch Laboratories, Inc., West Grove, PA, USA).

### Plasmids and DNA manipulations

Wild type A/Udorn/72 (H3N2 virus) NS1 gene (GenBank ID: V01102) was expressed in *E. coli* GST (pGEX-3X; Amersham Biosciences, Buckinghamshire, U. K.) and eukaryotic pcDNA3.1(+) (Invitrogen Corp., Carlsbad, CA, USA) expression vectors. Wild type A/WSN/33 (H1N1 virus) NS1 gene (GenBank ID: M12597) was modified by PCR to create N- and C-terminal *Bgl*II sites for further cloning into the *Bam*HI site of the pcDNA3.1(+) expression vector (Invitrogen). To create point mutations to the A/Udorn/72 and A/WSN/33 NS1 cDNAs, a QuikChange^TM^ Site-Directed Mutagenesis Kit (Stratagene, La Jolla, CA, USA) was used.

Wild type human nucleolin (C23; GenBank ID: BC002343), B23 (nucleophosmin; GenBank ID: BC050628), fibrillarin (FBL; GenBank ID: NM_001436.3) and HIV-1 Rev [48] genes were modified by PCR to create N- and C-terminal *Bam*HI sites for further cloning into the *Bam*HI site of the pcDNA3.1(+) expression vector (Invitrogen). Wild type human laminA/C (GenBank ID: NG_008692) gene was cloned into the pOTB7 vector (Invitrogen).

To create green fluorescence protein (GFP)-NS1 fusion constructs, N-terminal cDNAs for the wild type A/Udorn/72 (aa 1–73) and A/WSN/33 (aa 1–73) NS1 and C-terminal cDNAs (aa 157–237 and 157–230), respectively, and C-terminal cDNA for wild type A/Brevig Mission/1/18 (aa 203–230) genes were modified by PCR to create N- and C-terminal *Sal*I and *Nhe*I sites, respectively, for further cloning into the *Sal*I and *Nhe*I cloning sites of a pCMX-SAH/Y145F expression vector [49]. Mutations to the GFP-NS1 chimeric gene constructs were done using the QuikChange^TM^ Site-Directed Mutagenesis Kit (Stratagene). All oligonucleotides used to modify the genes in the study will be provided upon request.

All DNA manipulations were performed according to standard protocols, and the newly created gene constructs were partially or fully sequenced.

### NS1 binding assay, SDS-PAGE and Western blotting

Influenza A virus GST-NS1 fusion proteins were expressed in *E. coli* BL21 cells, and GST-fusion proteins were purified as described [37]. *In vitro*-translated nucleolin, B23 and fibrillarin wt proteins (TNT Coupled Reticulocyte Lysate Systems, Promega, Madison, WI, USA) were ^35^S]-labeled (PRO-MIX, Amersham Biosciences) and allowed to bind to Sepharose-immobilized GST or GST-NS1 fusion proteins on ice for 60 min followed by washing. GST-NS1 fusion protein-bound ^35^S]-labeled proteins were separated on 12% SDS-PAGE. The gels were fixed and treated with Amplify reagent (Amersham Biosciences) as specified by the manufacturer and autoradiographed. GST pull-down experiments from A549 cell extracts were carried out as described [50].

### Transfections, indirect immunofluorescence and confocal laser microscopy

For indirect immunofluorescence and confocal laser microscopy HuH7 cells, grown on glass coverslips for 24 h, were transfected with GFP, GFP-NS1 or HIV-1-pcDNA3.1(+) expression constructs using FuGENE6 transfection reagent (Roche Diagnostics, Indiapolis, IN, USA) according to the manufacturer’s instructions. Forty-eight hours after transfection the cells were fixed with 3% paraformaldehyde at RT for 20 min and processed for immunofluorescence microscopy. A549 cells were infected with influenza A/Udorn/72 wt virus for 5 to 8 hours as indicated in the legends for figures, fixed with 3% paraformaldehyde at RT for 20 min, permeabilized with 0.1% Triton X-100 for 5 min and processed for immunofluorescence microscopy. The cells, positive for transiently transfected GFP and GFP-NS1 or viral NS1 proteins, were visualized and photographed on a Leica TCS NT confocal microscope.

## Competing interest

The authors declare that they have no competing interests.

## Authors’ contributions

KM participated in the design of the study, performed most of the experiments, analyzed the results and drafted the manuscript. JT and RF participated in the design of the study and carried out some experiments. PR and DH-V provided crucial reagents to carry out the experiments and analyzed the confocal microscopy results. IJ initiated the study, participated in the design and coordination and helped to draft the manuscript. All authors have read and approved the final version of the manuscript.

## References

[B1] Jagger BW Wise HM Kash JC Walters KA Wills NM Xiao YL Dunfee RL Schwartzman LM Ozinsky A Bell GL An Overlapping Protein-Coding Region in Influenza A Virus Segment 3 Modulates the Host Response Science 2012 337 119 204 10.1126/science.1222213 PMC3552242 22745253

[B2] Kawaoka Y Gorman OT Ito T Wells K Donis RO Castrucci MR Donatelli I Webster RG Influence of host species on the evolution of the nonstructural (NS) gene of influenza A viruses Virus Res 1998 55 143 156 10.1016/S0168-1702(98)00038-0 9725667

[B3] Garten RJ Davis CT Russell CA Shu B Lindstrom S Balish A Sessions WM Xu X Skepner E Deyde V Antigenic and genetic characteristics of swine-origin 2009 A(H1N1) influenza viruses circulating in humans Science 2009 325 197 201 10.1126/science.1176225 19465683 PMC3250984

[B4] Min JY Krug RM The primary function of RNA binding by the influenza A virus NS1 protein in infected cells: Inhibiting the 2'-5' oligo (A) synthetase/RNase L pathway Proc Natl Acad Sci U S A 2006 103 7100 7105 10.1073/pnas.0602184103 16627618 PMC1459024

[B5] Mibayashi M Martinez-Sobrido L Loo YM Cardenas WB Gale M Jr Garcia-Sastre A Inhibition of retinoic acid-inducible gene I-mediated induction of beta interferon by the NS1 protein of influenza A virus J Virol 2007 81 514 524 10.1128/JVI.01265-06 17079289 PMC1797471

[B6] Pichlmair A Schulz O Tan CP Naslund TI Liljestrom P Weber F Reis e Sousa C RIG-I-mediated antiviral responses to single-stranded RNA bearing 5'-phosphates Science 2006 314 997 1001 10.1126/science.1132998 17038589

[B7] Kato H Takeuchi O Sato S Yoneyama M Yamamoto M Matsui K Uematsu S Jung A Kawai T Ishii KJ Differential roles of MDA5 and RIG-I helicases in the recognition of RNA viruses Nature 2006 441 101 105 10.1038/nature04734 16625202

[B8] Matikainen S Siren J Tissari J Veckman V Pirhonen J Severa M Sun Q Lin R Meri S Uze G Tumor necrosis factor alpha enhances influenza A virus-induced expression of antiviral cytokines by activating RIG-I gene expression J Virol 2006 80 3515 3522 10.1128/JVI.80.7.3515-3522.2006 16537619 PMC1440408

[B9] Siren J Imaizumi T Sarkar D Pietila T Noah DL Lin R Hiscott J Krug RM Fisher PB Julkunen I Matikainen S Retinoic acid inducible gene-I and mda-5 are involved in influenza A virus-induced expression of antiviral cytokines Microbes Infect 2006 8 2013 2020 10.1016/j.micinf.2006.02.028 16797201

[B10] Chen Z Li Y Krug RM Influenza A virus NS1 protein targets poly(A)-binding protein II of the cellular 3'-end processing machinery EMBO J 1999 18 2273 2283 10.1093/emboj/18.8.2273 10205180 PMC1171310

[B11] Li Y Chen ZY Wang W Baker CC Krug RM The 3'-end-processing factor CPSF is required for the splicing of single-intron pre-mRNAs in vivo RNA 2001 7 920 931 10.1017/S1355838201010226 11421366 PMC1370139

[B12] Nemeroff ME Barabino SM Li Y Keller W Krug RM Influenza virus NS1 protein interacts with the cellular 30 kDa subunit of CPSF and inhibits 3'end formation of cellular pre-mRNAs Mol Cell 1998 1 991 1000 10.1016/S1097-2765(00)80099-4 9651582

[B13] Noah DL Twu KY Krug RM Cellular antiviral responses against influenza A virus are countered at the posttranscriptional level by the viral NS1A protein via its binding to a cellular protein required for the 3' end processing of cellular pre-mRNAS Virology 2003 307 386 395 10.1016/S0042-6822(02)00127-7 12667806

[B14] Shimizu K Iguchi A Gomyou R Ono Y Influenza virus inhibits cleavage of the HSP70 pre-mRNAs at the polyadenylation site Virology 1999 254 213 219 10.1006/viro.1998.9555 9986787

[B15] Twu KY Noah DL Rao P Kuo RL Krug RM The CPSF30 binding site on the NS1A protein of influenza A virus is a potential antiviral target J Virol 2006 80 3957 3965 10.1128/JVI.80.8.3957-3965.2006 16571812 PMC1440456

[B16] Min JY Li S Sen GC Krug RM A site on the influenza A virus NS1 protein mediates both inhibition of PKR activation and temporal regulation of viral RNA synthesis Virology 2007 363 236 243 10.1016/j.virol.2007.01.038 17320139

[B17] Heikkinen LS Kazlauskas A Melen K Wagner R Ziegler T Julkunen I Saksela K Avian and 1918 Spanish influenza a virus NS1 proteins bind to Crk/CrkL Src homology 3 domains to activate host cell signaling J Biol Chem 2008 283 5719 5727 18165234 10.1074/jbc.M707195200

[B18] Gack MU Albrecht RA Urano T Inn KS Huang IC Carnero E Farzan M Inoue S Jung JU Garcia-Sastre A Influenza A virus NS1 targets the ubiquitin ligase TRIM25 to evade recognition by the host viral RNA sensor RIG-I Cell Host Microbe 2009 5 439 449 10.1016/j.chom.2009.04.006 19454348 PMC2737813

[B19] Li S Min JY Krug RM Sen GC Binding of the influenza A virus NS1 protein to PKR mediates the inhibition of its activation by either PACT or double-stranded RNA Virology 2006 349 13 21 10.1016/j.virol.2006.01.005 16466763

[B20] Bornholdt ZA Prasad BV X-ray structure of NS1 from a highly pathogenic H5N1 influenza virus Nature 2008 456 985 988 10.1038/nature07444 18987632 PMC2798118

[B21] Hale BG Batty IH Downes CP Randall RE Binding of influenza A virus NS1 protein to the inter-SH2 domain of p85 suggests a novel mechanism for phosphoinositide 3-kinase activation J Biol Chem 2008 283 1372 1380 18029356 10.1074/jbc.M708862200

[B22] Boisvert FM van Koningsbruggen S Navascues J Lamond AI The multifunctional nucleolus Nat Rev Mol Cell Biol 2007 8 574 585 10.1038/nrm2184 17519961

[B23] Hernandez-Verdun D Roussel P Thiry M Sirri V Lafontaine DL The nucleolus: structure/function relationship in RNA metabolism Wiley Interdiscip Rev RNA 2010 1 415 431 10.1002/wrna.39 21956940

[B24] Olson MO Sensing cellular stress: another new function for the nucleolus? Sci STKE 2004 2004 pe10 10.1126/stke.2242004pe10 15026578

[B25] Olson MO Hingorani K Szebeni A Conventional and nonconventional roles of the nucleolus Int Rev Cytol 2002 219 199 266 12211630 10.1016/S0074-7696(02)19014-0 PMC7133188

[B26] Pederson T Tsai RY In search of nonribosomal nucleolar protein function and regulation J Cell Biol 2009 184 771 776 10.1083/jcb.200812014 19289796 PMC2699146

[B27] Carmo-Fonseca M The contribution of nuclear compartmentalization to gene regulation Cell 2002 108 513 521 10.1016/S0092-8674(02)00650-5 11909522

[B28] Emmott E Hiscox JA Nucleolar targeting: the hub of the matter EMBO Rep 2009 10 231 238 10.1038/embor.2009.14 19229283 PMC2658561

[B29] Hiscox JA RNA viruses: hijacking the dynamic nucleolus Nat Rev Microbiol 2007 5 119 127 10.1038/nrmicro1597 17224921 PMC7097444

[B30] Olson MO Dundr M The moving parts of the nucleolus Histochem Cell Biol 2005 123 203 216 10.1007/s00418-005-0754-9 15742198

[B31] Quaye IK Toku S Tanaka T Sequence requirement for nucleolar localization of rat ribosomal protein L31 Eur J Cell Biol 1996 69 151 155 8907615

[B32] Macken C Lu H Goodman J Boykin L Osterhaus ADME, Cox N, Hampson AW The value of a database in surveillance and vaccine selection Options for the Control of Influenza IV 2001 Elsevier Science, Amsterdam 103 106

[B33] Timmers AC Stuger R Schaap PJ van 't Riet J Raue HA Nuclear and nucleolar localization of Saccharomyces cerevisiae ribosomal proteins S22 and S25 FEBS Lett 1999 452 335 340 10.1016/S0014-5793(99)00669-9 10386617

[B34] Birbach A Bailey ST Ghosh S Schmid JA Cytosolic, nuclear and nucleolar localization signals determine subcellular distribution and activity of the NF-kappaB inducing kinase NIK J Cell Sci 2004 117 3615 3624 10.1242/jcs.01224 15252129

[B35] Lechertier T Sirri V Hernandez-Verdun D Roussel P A B23-interacting sequence as a tool to visualize protein interactions in a cellular context J Cell Sci 2007 120 265 275 10.1242/jcs.03345 17179202

[B36] Sirri V Urcuqui-Inchima S Roussel P Hernandez-Verdun D Nucleolus: the fascinating nuclear body Histochem Cell Biol 2008 129 13 31 10.1007/s00418-007-0359-6 18046571 PMC2137947

[B37] Melen K Kinnunen L Fagerlund R Ikonen N Twu KY Krug RM Julkunen I Nuclear and nucleolar targeting of influenza A virus NS1 protein: striking differences between different virus subtypes J Virol 2007 81 5995 6006 10.1128/JVI.01714-06 17376915 PMC1900311

[B38] Murayama R Harada Y Shibata T Kuroda K Hayakawa S Shimizu K Tanaka T Influenza A virus non-structural protein 1 (NS1) interacts with cellular multifunctional protein nucleolin during infection Biochem Biophys Res Commun 2007 362 880 885 10.1016/j.bbrc.2007.08.091 17767916

[B39] Li Y Yamakita Y Krug RM Regulation of a nuclear export signal by an adjacent inhibitory sequence: the effector domain of the influenza virus NS1 protein Proc Natl Acad Sci U S A 1998 95 4864 4869 10.1073/pnas.95.9.4864 9560194 PMC20179

[B40] Cochrane AW Perkins A Rosen CA Identification of sequences important in the nucleolar localization of human immunodeficiency virus Rev: relevance of nucleolar localization to function J Virol 1990 64 881 885 2404140 10.1128/jvi.64.2.881-885.1990 PMC249184

[B41] Fankhauser C Izaurralde E Adachi Y Wingfield P Laemmli UK Specific complex of human immunodeficiency virus type 1 rev and nucleolar B23 proteins: dissociation by the Rev response element Mol Cell Biol 1991 11 2567 2575 2017166 10.1128/mcb.11.5.2567-2575.1991 PMC360026

[B42] Perkins A Cochrane AW Ruben SM Rosen CA Structural and functional characterization of the human immunodeficiency virus rev protein J Acquir Immune Defic Syndr 1989 2 256 263 2656990

[B43] Qin XF An DS Chen IS Baltimore D Inhibiting HIV-1 infection in human T cells by lentiviral-mediated delivery of small interfering RNA against CCR5 Proc Natl Acad Sci U S A 2003 100 183 188 10.1073/pnas.232688199 12518064 PMC140921

[B44] Nakabayashi H Taketa K Miyano K Yamane T Sato J Growth of human hepatoma cells lines with differentiated functions in chemically defined medium Cancer Res 1982 42 3858 3863 6286115

[B45] Neumann G Watanabe T Ito H Watanabe S Goto H Gao P Hughes M Perez DR Donis R Hoffmann E Generation of influenza A viruses entirely from cloned cDNAs Proc Natl Acad Sci U S A 1999 96 9345 9350 10.1073/pnas.96.16.9345 10430945 PMC17785

[B46] Gaush CR Smith TF Replication and plaque assay of influenza virus in an established line of canine kidney cells Appl Microbiol 1968 16 588 594 5647517 10.1128/am.16.4.588-594.1968 PMC547475

[B47] Malim MH Cullen BR HIV-1 structural gene expression requires the binding of multiple Rev monomers to the viral RRE: implications for HIV-1 latency Cell 1991 65 241 248 10.1016/0092-8674(91)90158-U 2015625

[B48] Malim MH Hauber J Fenrick R Cullen BR Immunodeficiency virus rev trans-activator modulates the expression of the viral regulatory genes Nature 1988 335 181 183 10.1038/335181a0 3412474

[B49] Ogawa H Inouye S Tsuji FI Yasuda K Umesono K Localization, trafficking, and temperature-dependence of the Aequorea green fluorescent protein in cultured vertebrate cells Proc Natl Acad Sci U S A 1995 92 11899 11903 10.1073/pnas.92.25.11899 8524871 PMC40510

[B50] Fagerlund R Kinnunen L Kohler M Julkunen I Melen K NF-{kappa}B is transported into the nucleus by importin {alpha}3 and importin {alpha}4 J Biol Chem 2005 280 15942 15951 10.1074/jbc.M500814200 15677444

